# Early Onset Severe Hypertensive Disease in Pregnancy and Screening for Antiphospholipid Syndrome

**DOI:** 10.1055/s-0040-1702926

**Published:** 2020-03-04

**Authors:** Nasim C. Sobhani, Rachel Shulman, Erin E. Tran, Juan M. Gonzalez

**Affiliations:** 1 Department of Obstetrics, Gynecology, and Reproductive Sciences, University of California San Francisco, San Francisco, California; 2 Georgia Perinatal Consultants, Atlanta, Georgia; 3 Department of Obstetrics and Gynecology, NorthShore University Health System, Evanston, Illinois

**Keywords:** antiphospholipid syndrome, preterm preeclampsia, antiphospholipid screening, severe hypertensive disease of pregnancy, antiphospholipid antibodies

## Abstract

**Objective** Although preterm delivery (PTD) before 34 weeks for severe hypertensive disease is a diagnostic criterion for antiphospholipid syndrome (APS), there is no consensus regarding testing for antiphospholipid antibodies (aPL) in this setting. We aim to describe the frequency of and the characteristics associated with inpatient aPL testing in this population.

**Study Design** In this retrospective study of PTD before 34 weeks for severe hypertensive disease, charts were reviewed for aPL testing, gestational age at delivery, fetal complications, and severity of maternal disease. Wilcoxon rank-sum test, Fisher's exact, and chi-squared tests were used for analyses of continuous and categorical variables, and multivariate logistic regression for adjusted odds ratios.

**Results** Among 133 cases, 14.3% had APS screening via aPL testing. Screened patients delivered earlier than unscreened patients (28.9 vs. 31.7 weeks, *p* <0.001). Each additional week of gestation was associated with a 39% decrease in the odds of screening (95% confidence interval: 0.43–0.85). There were no other differences between the groups.

**Conclusion** APS screening after PTD for severe hypertensive disease is uncommon but more likely with earlier PTD. Despite conflicting recommendations from professional organizations, prior studies demonstrate contraceptive, obstetrical, and long-term risks associated with APS, suggesting that we should increase our screening efforts.

Antiphospholipid syndrome (APS) is a multisystem autoimmune disease characterized by persistent antiphospholipid antibodies (aPL) plus thrombotic and/or obstetric complications. Severe hypertensive disease of pregnancy that requires delivery prior to 34 weeks has been part of the clinical diagnostic criteria for APS since the Sapporo criteria were published in 1999 and revised in 2006. 1 2 Laboratory diagnostic criteria include positive aPL on two occasions at least 12 weeks apart, with the initial testing typically performed at the time of the qualifying clinical event. For pregnant women undergoing iatrogenic preterm delivery before 34 weeks for severe hypertensive disease, the antepartum, intrapartum, and immediate postpartum periods are ideal opportunities to perform APS screening via laboratory testing for aPL. However, APS screening for women with early onset severe hypertensive disease of pregnancy is not yet routine, in large part due to conflicting recommendations from professional societies regarding screening this population and subsequent management of women identified with obstetric-only APS (i.e., those who have APS but no prior thromboembolic disease). 3 4 5 6 7 8 9 As a result, there is little understanding of how often screening occurs in this specific population or which clinical factors may prompt providers to perform this screening.

We designed this study to describe the frequency of inpatient antepartum, intrapartum, or immediate postpartum APS screening with aPL testing in women with early onset severe hypertensive disease in pregnancy and to identify clinical characteristics associated with increased likelihood of APS screening. We hypothesized that markers of more severe hypertensive disease (i.e., earlier gestational age at delivery, maternal symptoms, maternal laboratory abnormalities, and/or fetal complications) would be associated with an increased likelihood of aPL testing in this population.

## Methods

This was a retrospective cohort study that included all pregnancies with iatrogenic delivery prior to 34 weeks of gestation at the University of California, San Francisco (UCSF) from 2012 to 2017 for the following indications: preeclampsia with severe features, superimposed preeclampsia with severe features, hemolysis with elevated liver enzymes and low platelets (HELLP) syndrome, or eclampsia. These hypertensive diseases were diagnosed in accordance with the 2002 American College of Obstetricians and Gynecologists (ACOG) preeclampsia practice bulletin for patients who delivered between January 2012 and November 2013, and in accordance with the 2013 ACOG Task Force on Hypertension in Pregnancy for those who delivered after November 2013. 10 11 Pregnancies that ended in preterm delivery prior to 34 weeks for other indications (e.g., preterm labor, preterm premature rupture of membranes, or nonreassuring fetal status based on fetal heart tracing, ultrasound findings, or biophysical profile) were excluded. Subjects who had previously undergone aPL testing (in a prior pregnancy or in a nonpregnant state) were excluded, as were those with a known diagnosis of APS.

For all deliveries at UCSF, details regarding maternal demographics, labor characteristics, and pregnancy outcomes are collected at the time of delivery by the managing clinicians and stored within the UCSF Perinatal Database. Daily chart review is performed by trained abstractors to ensure accurate and complete information, while monthly review of the database is performed by trained physicians for quality assurance. We utilized this database to obtain information regarding hypertensive diagnosis, maternal demographics, pre-existing maternal medical conditions, fetal complications, and obstetrical outcomes for this cohort of women.

Because the UCSF Perinatal Database does not include information regarding laboratory testing or subjective report of symptoms, a separate chart review was performed by a single Maternal Fetal Medicine clinician (NCS) to obtain information regarding aPL testing, laboratory markers of more severe hypertensive disease (including thrombocytopenia defined as platelet count < 100,000/µL, transaminitis defined as aspartate aminotransferase ≥ 84 U/L and/or alanine aminotransferase ≥ 100 U/L, and acute kidney injury defined as creatinine > 1.1 mg/dL or double the patient's baseline), and symptoms of hypertensive disease (defined as severe headache, visual changes, right upper quadrant pain, and/or midepigastric pain).

Fisher's exact tests and chi-squared tests were used for analyses of categorical variables, *t* -tests, and Wilcoxon rank-sum test were used for analyses of continuous variables, and multivariate logistic regression was used to generate adjusted odds ratios. Statistical analyses were performed using STATA software (Version 14, College Station, TX) and SAS software (Version 9.4, Cary, NC). This study was approved by the UCSF Committee on Human Research (approval number 15–17358).

## Results

From 2012 to 2017, 133 pregnancies were complicated by severe hypertensive disease requiring iatrogenic delivery before 34 weeks. Nineteen (14.3%) pregnancies had APS screening via aPL testing. There were no statistically significant differences in maternal demographics, assisted reproductive technology, multiple gestation, or cesarean delivery between the two groups ( Table 1 ).

**Table 1:** Demographic comparison of women with iatrogenic preterm delivery before 34 weeks for severe hypertensive disease of pregnancy who did and did not undergo screening for antiphospholipid syndrome

	Unscreened ( *n* = 114)	Screened ( *n* = 19)	*p* -Value
Maternal age, years	31.6 (6.2)	29.1 (5.9)	0.09
Body mass index, kg/m ^2^	27.6 (17.9–76.3)	27.3 (19.1–50.0)	0.89
Race			
White	32 (28.1)	6 (31.6)	0.73
Black	13 (11.4)	2 (10.5)	
Latina	32 (28.1)	4 (21.1)	
Asian	20 (17.5)	2 (10.5)	
Other	17 (14.9)	5 (26.3)	
Education a			
Less than high school	9 (9.8)	1 (10.0)	0.58
High school	44 (47.8)	7 (70.0)	
College	32 (34.8)	2 (20.0)	
More than college	7 (7.6)	0 (0)	
Married	65 (57.0)	8 (42.1)	0.23
Insurance a			
Private	65 (58.0)	9 (50.0)	0.35
Medicaid	41 (36.6)	8 (44.4)	
Medicare	1 (0.9)	1 (5.6)	
Other	5 (4.5)	0 (0)	
Substance use	20 (17.5)	0 (0)	0.08
Nulliparous	48 (42.1)	5 (26.3)	0.22
Assisted reproductive technology	15 (13.2)	0 (0)	0.13
Multiple gestation	23 (20.2)	2 (10.5)	0.57
Cesarean delivery	76 (66.7)	13 (68.4)	0.88

a *n* = 92 for unscreened group and *n* = 10 for screened group for education; *n* = 112 for unscreened group and *n* = 18 for screened group for insurance, due to incomplete data.

Data are presented as *n* (%) for categorical variables, mean (standard deviation) for continuous variables with parametric distribution, and median (range) for continuous variables with nonparametric distribution.

Obstetrical, maternal, and fetal complications are summarized in Table 2 . Compared with unscreened women, screened women delivered at a significantly earlier gestational age, with median gestational age of 28.9 (range: 24.9–33.7) weeks compared with 31.7 (range: 23.1–33.9) weeks ( *p* < 0.001). In multivariate logistic regression controlling for numerous maternal demographics (i.e., maternal age, race, body mass index, education, marital status, and insurance), each additional week of gestation was associated with a 39% decrease in the odds of screening (adjusted odds ratio 0.61, 95% confidence interval: 0.43–0.85, *p* = 0.004). There were no differences in specific hypertensive diagnosis, severity of maternal disease (as measured by symptoms and/or laboratory abnormalities), or pre-existing maternal complications between the two groups. There were similarly no differences between the two groups with regard to fetal complications, including growth restriction, oligohydramnios, and absent end diastolic flow on umbilical artery Doppler interrogation.

**Table 2:** Comparison of women with iatrogenic preterm delivery before 34 weeks for severe hypertensive disease of pregnancy who did and did not undergo screening for antiphospholipid syndrome

	Unscreened ( *n* = 114)	Screened ( *n* = 19)	*p-* Value
Gestational age at delivery, weeks	31.7 (23.1–33.9)	28.9 (24.9–33.7)	< 0.001
Diagnosis			
Severe preeclampsia	57 (50.0)	9 (47.4)	0.63
Superimposed preeclampsia	32 (28.1)	4 (21.1)	
HELLP syndrome	22 (19.3)	5 (26.3)	
Eclampsia	3 (2.6)	1 (5.3)	
Symptoms	60 (52.6)	7 (36.8)	0.20
Thrombocytopenia a	26 (22.8)	5 (27.8)	0.77
Transaminitis a	50 (43.9)	10 (16.7)	0.35
Acute kidney injury a	21 (18.4)	1 (5.6)	0.31
Prior hypertensive disease	35 (30.7)	4 (21.1)	0.59
Prior renal disease a	3 (2.7)	1 (5.3)	0.47
Prior thromboembolic disease	3 (2.6)	0 (0)	1.00
Fetal growth restriction	22 (19.3)	4 (21.1)	1.00
Oligohydramnios a	6 (5.9)	2 (11.8)	0.32
Absent end diastolic flow in umbilical artery	4 (3.5)	1 (5.3)	0.54

Abbreviations: ALT, alanine aminotransferase; AST, aspartate aminotransferase; HELLP, hemolysis with elevated liver enzymes and low platelets.

a *n* = 18 for screened group for thrombocytopenia, transaminitis, acute kidney injury; *n* = 113 for unscreened group for prior renal disease; and *n* = 102 for unscreened group and *n* = 17 for screened group for oligohydramnios, due to incomplete data in electronic medical record.

Thrombocytopenia was defined as platelet count < 100,000/µL, transaminitis as AST ≥ 84 U/L and/or ALT ≥ 100 U/L, and acute kidney injury as creatinine > 1.1 mg/dL or double the patient's baseline.

Data are presented as *n* (%) for categorical variables and median (range) for continuous variables with nonparametric distribution.

## Discussion

Less than 15% of women with severe hypertensive disease of pregnancy requiring delivery before 34 weeks undergo APS screening during the antepartum, intrapartum, or immediate postpartum periods. We were surprised to find that so few patients were screened at the time of a qualifying clinical diagnosis for this serious syndrome. We did find an increased likelihood of APS screening in pregnancies that delivered at earlier gestational ages, and we suspect that earlier gestational age at delivery suggests particularly severe hypertensive disease and serves as a reminder to screen for APS. Screening was otherwise not associated with any other markers of severity of hypertensive disease, including maternal or fetal complications.

This study is limited by its small sample size, which affects the ability to detect small differences in rare outcomes; by its retrospective design, which limits data collection to what has been previously captured in the electronic medical record; and by its inclusion of a single institution, which limits generalizability of results. Furthermore, this study only examined inpatient testing for aPL and thus may have missed patients who underwent aPL testing as in the outpatient setting after postpartum discharge from the hospital. Because patients were initially diagnosed at a tertiary care center that serves as a referral center for a wide catchment area, we were not able to evaluate for outpatient aPL testing, as many patients returned to external providers for post-discharge medical care. Despite this limitation, focusing specifically on aPL testing in the inpatient setting immediately after the qualifying clinical event remains important; this is the ideal time to perform initial screening, because it reduces the possibility of missed diagnoses due to loss to follow-up or due to transfer of medical care with inadequate or incomplete transfer of medical records.

The major strength of this study is the demonstration of infrequent APS screening in a population that meets clinical criteria for a syndrome associated with long-term obstetric and thrombotic risks. Infrequent APS screening in this population may be due to conflicting recommendations from obstetric and nonobstetric professional societies regarding management of these patients ( Table 3 ). While the Royal College of Obstetricians and Gynaecologists (RCOG) and the Society of Obstetricians and Gynaecologists of Canada (SOGC) do not address the question of APS screening for women with early onset severe hypertensive disease of pregnancy, the Royal Australian and New Zealand College of Obstetricians and Gynaecologists (RANZCOG) maintains that this condition is a definitive indication for APS screening. 4 7 9 In contrast, ACOG recommends against routine aPL testing in this population. 3

**Table 3:** Recommendations from obstetric professional societies regarding screening for APS and management of obstetric-only APS

	ACOG	RCOG	SOGC	RANZCOG
Should women with early onset severe hypertensive disease of pregnancy be screened for APS with aPL testing?	No	Not addressed	Not addressed	Yes
Should women with APS but no prior thrombotic history receive postpartum thromboprophylaxis?	Consider	Consider	Yes	Not addressed
Should women with APS but no prior thrombotic history use estrogen-containing contraceptives?	Avoid	Contraindicated	Not addressed	Avoid

Abbreviations: ACOG, American College of Obstetricians and Gynecologists; aPL, antiphospholipid antibodies; APS, antiphospholipid syndrome; RANZCOG, Royal Australian and New Zealand College of Obstetricians and Gynaecologists; RCOG, Royal College of Obstetricians and Gynaecologists; SOGC, Society of Obstetricians and Gynaecologists of Canada.

Because women with APS require specialized counseling and management to reduce thrombotic and obstetric risks, providers who do not screen this population for APS may be missing a critical opportunity to detect the presence of and alter the course of a serious disease. Women with obstetric-only APS carry an increased risk of thromboembolic disease despite an absence of prior thromboembolic disease. 12 13 To minimize this risk in the hypercoagulable postpartum period, SOGC and the British Committee for Standards in Hematology (BSCH) recommend that postpartum thromboprophylaxis be given to any woman with APS, while ACOG and RCOG recommend that postpartum thromboprophylaxis at least be considered for this population. 3 5 8 14 Similarly, ACOG, RANZCOG, and the European League Against Rheumatism (EULAR) recommend that women with obstetric-only APS avoid estrogen-containing contraceptives, while RCOG states more strongly that estrogen-containing contraceptives are frankly contraindicated in this population. 3 6 9 15

With regard to reducing obstetric risks, there is currently insufficient evidence that treatment in subsequent pregnancies reduces the risk of late-pregnancy complications in women with APS, with most prior studies focusing on anticoagulation with heparins. 16 Newer studies, however, have suggested that other (i.e., nonheparin) therapeutics may be beneficial in this population. 17 For example, observational studies have demonstrated reduced rates of early onset severe hypertensive disease, intrauterine growth restriction, and other placenta-mediated complications when women with APS use hydroxychloroquine in pregnancy. 18 19 There is an ongoing randomized, placebo-controlled trial examining hydroxychloroquine for this indication; if the final results of this study are consistent with those of prior observational studies, there may be a shift in screening and management of this population. 20

We found that only a small minority of women with severe early onset hypertensive disease of pregnancy are being screened for APS via aPL testing in the inpatient setting at the time of initial diagnosis and that those who deliver at earlier gestational ages are more likely to undergo screening. Despite the known obstetric and thrombotic complications of APS, there is little consensus regarding screening for this disease and for managing women with obstetric-only APS. Review of consensus opinions and emerging studies suggest that consideration should be given to changing the current screening recommendations to better identify, risk-stratify, and manage women with APS.
